# The Effects of Transcranial Direct Current Stimulation During Extended Reality Exercises for Cortical, Neuromuscular, and Clinical Recovery of Stroke Survivors

**DOI:** 10.1155/np/5688648

**Published:** 2025-07-15

**Authors:** Cassio V. Ruas, Bruna M. Carlos, Saulo Feitosa, Márcio Vinícius Silva, Pedro Vazquez, Larissa L. Pontes, Jayne Silvestre, Sara R. M. Almeida, Alexandre F. Brandão, Gabriela Castellano

**Affiliations:** ^1^Brazilian Institute of Neuroscience and Neurotechnology (BRAINN), University of Campinas (UNICAMP), São Paulo, Campinas, Brazil; ^2^Institute of Physics Gleb Wataghin, University of Campinas (UNICAMP), São Paulo, Campinas, Brazil; ^3^School of Medical and Health Sciences, Edith Cowan University (ECU), Joondalup, Western Australia, Australia; ^4^Polytechnic School, Pontifical Catholic University of Campinas (PUC-Campinas), São Paulo, Campinas, Brazil

**Keywords:** electroencephalography, electromyography activity, extended reality, functional recovery, transcranial direct current stimulation

## Abstract

**Background:** Rehabilitation methods that include anodal transcranial direct current stimulation (*a*tDCS) and extended reality (XR) exercises have been used to enhance neural networks and improve functional performance in stroke patients, but the neuromuscular and neurophysiological mechanisms underlying these improvements are not fully understood. The purpose of this study was to examine the effects of *a*tDCS during XR rehabilitation exercises on cortical, neuromuscular, and clinical outcomes of stroke survivors.

**Methods:** Nineteen chronic stroke survivors were placed into either a transcranial direct current stimulation (tDCS) or a *Sham* group, without significant (*p* > 0.73) differences in the baseline levels of disability between groups. The tDCS group received active *a*tDCS and the *Sham* group received sham *a*tDCS applied on the ipsilesional primary motor cortex (M1) while performing a 10-session XR rehabilitation program. Surface electromyography (EMG) activity was recorded from deltoid and rectus femoris of the paretic limb without and with the application of active/sham *a*tDCS on the M1. Shoulder abduction and hip flexion active maximum joint range of motion (ROM_max_), electroencephalography (EEG)-derived brain symmetry index (BSI) and functional/clinical tests were assessed before and after the rehabilitation program.

**Results:** EMG activity was ~ 31% greater during hip flexion of the paretic limb with the application of active *a*tDCS than without *a*tDCS (*p*=0.04). Paretic hip flexion ROM_max_ increased by ~ 26%, BSI decreased by ~ 72% (indicating greater brain symmetry) and timed up and go (TUG) functional test improved by ~ 11% from before to after the rehabilitation program for the tDCS group only (*p* < 0.05). No other significant differences (*p* > 0.05) were observed.

**Conclusion:** It seems that the application of active *a*tDCS targeted the ipsilesional M1 representation of the quadriceps, which potentiated muscle activation in the paretic rectus femoris during XR exercises and resulted in greater motor recovery in hip flexion movements. The EEG-derived BSI results also indicate that *a*tDCS was effective in reorganizing the ipsilesional hemisphere brain activity after stroke.

## 1. Introduction

Stroke is one of the leading causes of disability worldwide. Over the last two decades, the absolute number of stroke incidents increased by 70% and the number of deaths from stroke increased by 43%, leading to a current estimated global cost of more than US$891 billion [1, 2]. The main risk factors contributing to stroke death and disability combined are high body mass index, high systolic blood pressure, high fasting plasma glucose, ambient particulate matter pollution, and smoking [2]. Previous prospective studies have also shown that reduced levels of physical activity and increased sedentary behavior (e.g., daily screen time for more than 4 h) are associated with a higher risk of new and recurrent episodes of stroke [3, 4]. Although stroke prevention measures and acute management of this condition seem to have progressively improved over the years, the burden of stroke is predicted to increase even more in the coming years [1, 2, 5]. As a result of the progressive care provision, a decrease in stroke death rate is expected to occur, which will in turn increase the number of stroke individuals surviving with a disability [6].

Hemiparesis is a common manifestation after a stroke episode, which may result in muscle weakness of the body in the contralateral side of the cortical lesion [6, 7]. This condition seems to be caused by reduced signal transmissions along descending neural pathways, affecting voluntary movement, motor control, and muscle function of stroke survivors [8]. In particular, maximum joint range of motion (ROM_max_) impairments in the paretic side of the body (limbs affected by hemiparesis) may occur after stroke and are often associated with increased pain and reduced motor function [6, 9, 10]. These impairments may develop early after a stroke episode and further aggravate with the lack of rehabilitation exercises [6, 9–11]. Furthermore, abnormal levels of surface electromyographic (EMG) activity are often reported in the paretic muscles of chronic stroke survivors [12, 13], which result from disorganization in the motor unit (MU) pool recruitment following a cortical lesion, potentially due to the early recruitment of larger and more fatigable MUs [13]. Thus, the modifications seen in both ROM_max_ and muscle activation levels seem to contribute to stroke-induced motor impairment and compromise muscle function [11, 13].

Stroke can also lead to impairment-specific changes with decreased neuronal activity near the cortical lesion, which may result in asymmetry between the affected and unaffected hemispheres of the brain [14–16]. In particular, electroencephalography (EEG) is a valuable tool to measure functional cortical activity, which provides insights into the reorganization of neuronal assembles in the brain after a stroke episode [15, 17]. EEG features have been extensively used for stroke evaluation, diagnosis, and even prognosis [17]. A common EEG parameter for assessing neurological function of stroke patients is the brain symmetry index (BSI), which was specifically developed for this population [15, 16]. The BSI compares spectral activity between the left and right hemispheres of the brain, indicating their level of similarity [16, 18]. Previous studies have reported that stroke survivors have more asymmetry between the two hemispheres compared to healthy controls [14], and that a greater BSI (which indicates greater asymmetry) is associated with an increased level of impairment (measured by Fugl-Meyer Assessment) [19]. However, whether effective neurorehabilitation therapies can induce plastic structural brain changes within the affected hemisphere and reduce cortical interhemispheric asymmetry after stroke is not fully understood.

Since stroke leads to modifications in neuroplasticity and synaptic organization, emerging neurorehabilitation methods have been proposed to enhance neural networks and reduce functional impairments in stroke survivors [20, 21]. For instance, both transcranial direct current stimulation (tDCS) and extended reality (XR) technologies have been used independently as rehabilitation methods for inducing motor recovery in neurological populations, including stroke [20, 22, 23]. Anodal tDCS (*a*tDCS) is a noninvasive brain stimulation technique, in which low-intensity electrical current is applied over the ipsilesional primary motor cortex (M1) to facilitate neuroplasticity by increasing corticomotor excitability [23, 24]. It has been previously shown that this technique may enhance brain activity, leading to greater improvements in motor learning and motor function after stroke [21, 25]. XR, a general term that encompasses both virtual and augmented reality, is a computer-based technology that allows users to interact with a simulated artificial environment and receive real-time feedback while performing exercises which activate the motor cortex and induce plastic changes in the brain [26–28]. XR has been used as a rehabilitation tool for improving motor skills while also allowing clinicians and researchers to gather precise kinematic and kinetic outcome measures of body movements in stroke survivors [22].

Some studies have also investigated the effects of combining both XR and *a*tDCS together during rehabilitation exercise programs for targeting improvements in motor performance and functionality in stroke survivors. For example, Llorens et al. [21] found that a rehabilitation intervention for stroke survivors that combined XR and *a*tDCS resulted in greater improvements in upper limb function compared to a conventional physical therapy program, while similar effects between interventions were found for sensory function (measured by clinical scales). In contrast, Viana et al. [28] reported that the addition of active *a*tDCS to an XR therapy after stroke resulted in greater improvements in wrist spasticity but similar motor function outcomes compared to XR paired with sham *a*tDCS. Similarly, a recent meta-analysis showed that the combination of tDCS (cathodal or anodal) along with XR therapy does not provide greater improvements in upper extremity function compared to XR alone, although the use of these combined rehabilitation techniques may improve the quality of life of stroke patients. However, to the best of our knowledge, the effects of *a*tDCS applied during XR exercises on cortical and neuromuscular outcomes of stroke survivors (including brain symmetry, upper and lower-limb motion analyses and muscle activation objective measures) have not been previously investigated.

Therefore, the aim of the present study was to examine the specific effects of *a*tDCS applied during XR rehabilitation exercises on EEG-derived BSI, active joint ROM of upper and lower extremities, and clinical/functional performance outcomes of stroke survivors. We also examined whether stroke individuals who received the application of active *a*tDCS on the M1 produced greater EMG activity compared to individuals who did not receive *a*tDCS. It was hypothesized that the application of *a*tDCS on the ipsilesional M1 would boost muscle activation of the rectus femoris and shoulder deltoid muscles. We also hypothesized that a 2-week rehabilitation program including *a*tDCS combined with XR exercises would improve interhemispheric brain symmetry and lead to greater upper- and lower-limb active joint ROM along with clinical and functional performance in stroke survivors.

## 2. Materials and Methods

### 2.1. Participants

A total of 19 chronic stroke survivors (9 males and 10 females; age: 58.9 ± 13.3 y) were recruited for the study. Recruitment of participants occurred by advertising the study (i.e., flyers, posters, and social media) in the local community, and with the assistance from the staff of the University's Clinical Hospital who provided a list of stroke patients in ambulatory care. The inclusion criteria of the study were: (1) previous occurrence of ischemic stroke event; (2) time since stroke event greater than 6 months; (3) hemiparesis of upper and lower limbs in the right or left extremities of the body, defined by upper and lower extremity subscale of the Fugl-Meyer Assessment [29–31]; (4) no brain or metallic implants in any part of the body; and (5) no use of heart pacemakers. The sample size was based on a previous study [21] reporting changes in motor function (assessed by the Fugl-Meyer Assessment Scale) from before (9.50 ± 5.11) to after (14.79 ± 7.37) a rehabilitation program consisting of *a*tDCS and VR in chronic stroke patients, which provided the Cohen's effect size of 1.2. Using G*⁣*^*∗*^Power 3.1 (Institute for Experimental Psychology, Dusseldorf, Germany), with a power of 0.8 and a significance level of 0.05, the required sample size was estimated to be 18 (9 per group). Accounting for an estimation error and potential dropouts, a total of 21 participants were recruited initially, but one participant decided to not continue in the study after the first session and another one dropped out after completing only 1 week of the rehabilitation program (5 sessions). Thus, the analyses were based on 19 participants who completed all the 10 training sessions. None of them were engaged in any external structured exercise/training program during the participation in the study.

This clinical trial was registered (RBR-89w7hc2) and followed the CONSORT checklist [32]. Participants were allocated to one of two parallel groups in a block randomized-controlled fashion: a tDCS group and a *Sham* group. As shown in Table 1, the two groups did not have any significant or discernable differences in age, months post-stroke, level of disability (measured by the Modified Rankin Scale), and extremities affected by hemiparesis after stroke. All participants were first informed about the risks and benefits of participating in the study. They also provided a written informed consent and completed a pre-exercise medical questionnaire to ensure they were free of neuromuscular injuries and/or illnesses, and to reduce any health or accident risks upon participation in the study. The questionnaire also accounted for any potential risks of receiving transcranial stimulation, such as previous episodes of epilepsy, convulsion, head trauma, or seizure, which would preclude the participation in the study [33]. In addition, the Modified Ranking Scale was used to assess any limitations in activity and changes in lifestyle of participants after the stroke episode [34]. Ethical approval was obtained from the University of Campinas Human Research Ethics Committee (Project no. 35771314.4.0000.5404) and all the procedures of the study were in accordance with the standards of the Declaration of Helsinki.

### 2.2. Experimental Design

**Figure 1:**
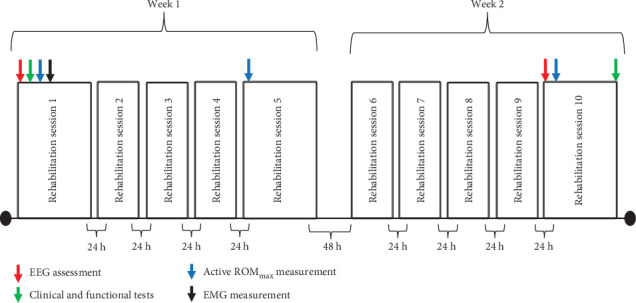
Study design indicating the order of neuromuscular and clinical measures assessed prior and/or after sessions one (baseline), five (mid-rehabilitation) and 10 (post-rehabilitation). Abbreviations: EEG, Electroencephalography; EMG, electromyographic activity; ROM_max_, active maximum range of motion.

All participants performed a structured 10-session full body rehabilitation program (30 min/session) consisting of XR exercises targeted for motor improvements in both paretic and nonparetic limbs (details explained below). Neuromuscular and clinical measures were assessed in the first rehabilitation session (baseline), and/or in sessions 5 (mid-rehabilitation) and 10 (post-rehabilitation). Prior and/or after each one of these sessions, the following assessments were completed: (1) EMG activity of the rectus femoris and shoulder deltoid during sets of upper and lower body maximal voluntary isometric contraction (MVIC) in session one; (2) Resting-state EEG for BSI measurement in sessions one and 10; (3) Shoulder abduction and hip flexion active ROM_max_ assessment in sessions one, five, and 10; and (4) Functional (Time up and Go [TUG] and 10-m walk) and clinical (Berg Balance Scale and Fugl-Meyer) assessments in session one and 10 of the rehabilitation program. Five- to 10-min intervals were given between assessments prior to sessions one and 10. One participant from the tDCS group did not complete the EMG and ROM_max_ tests. A diagram of the study design is shown in Figure 1.

### 2.3. Rehabilitation Program

**Figure 2:**
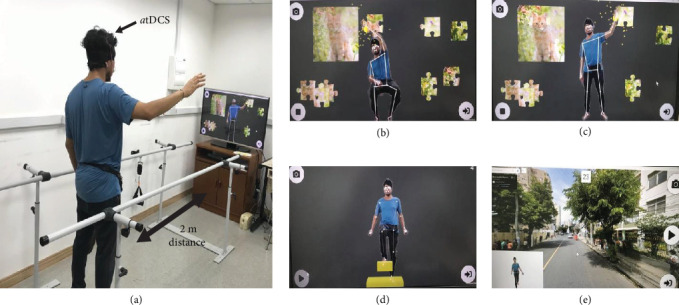
Set-up of rehabilitation exercises utilizing extended reality (XR, a). Jigsaw puzzle exercise (XR “GesturePuzzle” interactive game) performed sitting upright on a chair (b) and standing up on the floor or on top of an unstable surface (c). Step over obstacles exercise (XR “Obstacles” interactive game, d), and stationary walk exercise while visualizing an interactive Google Maps street view environment (XR “GestureMaps” game, e).

The rehabilitation program consisted of daily sessions occurring five times a week (30 min/session) for 2 weeks, with each weekly session separated by 24 h (total of 10 sessions). The rehabilitation program was divided into two blocks of 5 daily sessions with 48 h interval between them (Figure 1). In each session, participants interacted with a GestureCollection XR software [35] which displayed their images on a large television screen and created a simulated artificial environment. As shown in Figure 2a, participants were positioned at a 2-m distance facing the screen and performed three exercises for the upper limbs and two exercises for the lower limbs, respectively.

For the upper limbs, participants completed a jigsaw puzzle exercise (XR “GesturePuzzle” interactive game) by selecting puzzle pieces on one side of the screen and then fitting them together to form a complete picture frame. As shown in Figure 2b,c, participants performed this task in three different positions (which represented three different exercises): (1) Sitting upright on a chair; (2) Standing up; and (3) Standing up on top of an unstable surface (i.e., matt or stability disk). For each exercise, participants were required to complete three trials with the paretic (affected) upper limb and one trial with the nonparetic limb. No time limit was given for participants to complete these tasks.

For the lower limbs, participants performed two different exercises which involved walking in a stationary position, in the following sequence: (1) Raising hips and knees as high as possible to step over virtual obstacles with the paretic followed by nonparetic lower limb (XR “Obstacles” interactive game). For this exercise, participants were required to complete three trials of 1 min each (see Figure 2d). (2) Stationary walk while visualizing an interactive Google Map's street view (XR “GestureMaps” game) environment (i.e., city, area, route), which moved as participants raised their paretic and nonparetic hips and knees. This exercise consisted of one trial of 5 min (see Figure 2e).

For all exercises, ~30 s of rest were given between trials, and 1-min rest was given between exercises. All participants were familiarized with the rehabilitation protocol at the beginning of the first session, and the sessions were systematically supervised by trained instructors (physiotherapists and physical educators). During the rehabilitation program, the instructors introduced small progressions in the sessions as participants got used to the exercises, such as spreading the puzzle pieces farther apart on the screen for participants to reach at a greater shoulder abduction/flexion angle in the jigsaw puzzle exercises and increasing the threshold angle for participants to reach a greater hip/knee ROM when stepping over the obstacles or walking in the Google Map's street view virtual environment.

While completing the exercises, the tDCS group received active *a*tDCS and the *Sham* group received sham *a*tDCS, respectively, on the ipsilesional primary motor cortex (M1) for the same 30 min/session (details explained in section below).

### 2.4. tDCS

Low-intensity (2 mA) active *a*tDCS (Microestim NKL, Brusque, Brazil) was delivered using cathode and anode electrodes placed over the cortex of the patients from the tDCS group for the duration of each rehabilitation session (30 min/session). The anode was placed over the ipsilesional M1 (C3 or C4) and the cathode was placed at the contralesional supraorbital area of the cortex (Fp1 or Fp2) [21]. Prior to stimulation, the electrodes were fitted inside saline-soaked (i.e., 0.9% NaCl) sponges of 5 cm × 7 cm size to reduce the impedance, which was kept at *Z* < 40 kΩ.

The same settings were applied over the cortex of the participants of the *Sham* group, but for this group the *a*tDCS intensity rapidly ramped up to 2 mA in the first 30 s and then ramped down to 0 mA during the following 30 s, remaining at this value (sham stimulation) throughout the entire duration of each rehabilitation session [27]. The principal researcher administered the *a*tDCS configuration for each group. The remaining testers and instructors (who conducted the training sessions) and participants were blinded towards the stimulation parameters and group allocations.

### 2.5. EEG Recording

Resting EEG measurements were performed at sessions one and 10 of the rehabilitation program with participants seated upright and relaxed on a comfortable chair with their eyes open. Raw EEG signals were recorded at a sampling rate of 256 Hz in resting state using 16 dry electrodes (g.tec g.USBamp) positioned according to the 10/20 system (at the following locations: AFz, F1, F2, F5, F6, Cz, C1, C2, C3, C4, C5, C6, P1, P2, P5, P6), and a reference electrode positioned on the left mastoid. Each EEG recording lasted for 64 s. Signals were first filtered with an analog band-pass filter (0.1–100 Hz) and a notch filter at 60 Hz using the g.USBamp amplifier functions. Further processing included artifact removal with the HAPPILEE pipeline [36] and re-referencing using rCAR [37]. Data from one participant from the *a*tDCS group and one from the *Sham* group were excluded due to poor signal quality.

To compute BSI, the first and last 2 s of each acquisition were excluded, resulting in a total of 60 s of data. This data was then segmented into 10 6-s epochs. For each epoch, BSI was calculated across the delta (1–4 Hz), theta (4–8 Hz), alpha (8–13 Hz), beta (13–30 Hz), and gamma (30–40 Hz) frequency bands, and also for the entire spectrum (1–40 Hz). BSI calculations (ranges from 0 to 1) were performed according to the methods defined by van Putten [16], in which 0 = perfect symmetry between hemispheres, while 1 = complete asymmetry between hemispheres. The 10 BSI values from each acquisition were averaged to obtain one value per session and frequency band.

### 2.6. EMG Activity

Surface EMG activity from the quadriceps rectus femoris and shoulder medial deltoid of the paretic limbs was recorded in the baseline session by a Neuro-EMG-Micro-4 system (Neurosoft, Ivanovo, Russia). For the rectus femoris, one electrode was placed at ~ 50% of the line between the superior iliac spine and the superior part of the patella and the second, 5 cm distal, and a ground electrode was placed over the tibial tuberosity of the lower limb tested. For the medial deltoid, one electrode was placed at the greatest bulge of the muscle from the acromion to the lateral epicondyle of the elbow and the second, 5 cm distal, and a ground electrode was placed over the top outer edge of the shoulder acromion. The anatomical sites for electrode placement were selected in accordance to the “Surface EMG for Non-Invasive Assessment of Muscles (SENIAM) guidelines” [38] and a pseudo-monopolar orientation was used for the inter-electrode distance [39, 40]. The skin areas were shaved, abraded, and cleaned with 70% isopropyl alcohol swabs for electrode placement and to reduce impedance (*Z* < 5 kΩ).

Rectus femoris and medial deltoid EMG were recorded during hip flexion and knee extension, and shoulder abduction and shoulder flexion MVICs of the paretic limbs. The MVICs consisted of three 3-s maximum attempts for each joint movement. For the shoulder abduction and flexion MVICs, the participants were required to maintain their paretic shoulder parallel to the floor at a 90° angle (0° = full shoulder adduction/extension). For the knee extension and hip flexion MVICs, participants also maintained their paretic knee extended and hip flexed, respectively, at a 90° angle each (0° = full knee flexion/hip extension). However, some participants were not able to reach these angles due to the severity of their hemiparesis, so they performed the movement at their maximum possible joint ROM.

Since we wanted to examine the effects of active/sham *a*tDCS on EMG activity, participants from both groups repeated the same sequence of MVICs twice. The tDCS group performed the MVICs for each joint movement first without the application of *a*tDCS on the M1, followed by with the application of active *a*tDCS on the M1 (active stimulation). The *Sham* group also performed the same sequence of MVICs twice (without and with the application of *a*tDCS). However, for this group the first sequence of MVICs was performed without the application of *a*tDCS on the M1, while the second sequence was performed with the application of sham *a*tDCS on the M1 (i.e., sham stimulation). Participants from both groups remained blinded to the stimulation configurations of the tDCS condition that they were receiving.

A Neuro-MEP.NETω software (Neurosoft, Ivanovo, Russia) was used to record EMG signals at a sampling rate of 2-kHz (common mode rejection ratio >85 dB, gain = 1000). Raw EMG signals were band pass filtered (20–1000 Hz) and amplified (1000x). Signals were further analyzed offline using a LabChart software (v8, A ADInstruments, Bella Vista, NSW, Australia) and the root mean square (RMS) EMG was calculated over a 250 ms period during the highest MVICs. No further normalization of the EMG data was performed (i.e., by the means of MVIC or peak EMG) as this approach is often not recommended in people with stroke or other neurological conditions because of their reduced muscle strength as a consequence of paresis and/or increased spasticity, which can in turn affect the interpretation of the EMG (RMS) results [41, 42].

### 2.7. Maximum Joint ROM_max_ Assessment

Paretic and nonparetic active ROM_max_ of participants were measured during bilateral shoulder abduction and alternated hip flexion movements in sessions 1 (baseline), 5 (mid-session of rehabilitation) and 10 (last day of rehabilitation). Participants were positioned at a 2-m distance facing a large television screen which displayed their whole body image. The television was connected to a Kinect device system (v2, Microsoft, Albuquerque, NM, USA), which captured their joint movements through a grid of infrared dots at a sampling rate of 30 frames/second [43]. Participants performed three repetitions for each movement in which they were instructed to actively reach their ROM_max_.

Three attempts were given for participants to reach their active ROM_max_ for each joint movement. The ROM_max_ reached by the participants for the paretic and non-paretic shoulder abduction and hip flexion movements was recorded using a KinesiOS/Tracker software (Kinect One, v1.0.12, São Paulo, SP, Brazil) and further analyzed offline using Excel (v16.26, Microsoft, Albuquerque, NM, USA). For each joint movement (shoulder abduction and hip flexion), three values of paretic and nonparetic ROM_max_ were averaged and used for further comparisons.

### 2.8. Clinical and Functional Tests

All participants were assessed for clinical and functional tests before and after the rehabilitation program by an experienced evaluator. Clinical outcomes were assessed by the Fugl-Meyer Assessment and the Berg Balance Scales.

The Fugl-Meyer is designed to assess motor function and balance, sensation, passive ROM and occurrence of joint pain and includes three assessment grades (0 as minimum score and 2 as maximum score) for each item of the scale, totalizing a maximum of 226 points [44]. Participants were assessed for the full Fugl Meyer test (total score) and both upper-extremity (maximum score = 66) and lower-extremity (maximum = 34) Fugl-Meyer motor subscales [29, 30]. The Berg Balance Scale is used to assess the ability of individuals to balance and is composed of a scale from 0 to 56 points in 14 common balance items in everyday life [45].

Participants also performed the timed up and go (TUG) and 10-m walk functional tests. For TUG, participants started the test seated upright on a comfortable chair of 45 cm height and without handles. As authorized by the researcher they stood up and walked straight for 3 m, turned around a cone, walked back and sat down on the chair again [46]. For the 10-m walk, participants started in a standing position and walked straight for 10 m until reaching a finish line mark (determined by a cone on the floor) [47, 48]. Participants were instructed to perform all tests as fast as possible. The fastest times in seconds (recorded by a stopwatch) that the participants took to complete each test were taken and used for further analyses.

### 2.9. Statistical Analyses

Data were first screened for normality of distribution and homogeneity of variances using the Shapiro-Wilk test and the Levene's test, respectively. Since most of the data were deemed without a normal distribution and/or had a high inter-individual variability, nonparametric tests and linear mixed models were used to compare the dependent variables over time for each group. Shoulder abduction and hip flexion ROM_max_ values measured in sessions one, five, and 10 of the rehabilitation program were compared using linear mixed models with a repeated measures design. The use of linear mixed models with a repeated measures design allows grouping of individuals and/or variables from different units of measurement, reducing inter-individual variability and eliminating confounding variables when changes from before to after interventions are compared [46, 49, 50]. The most appropriate covariance structure of the model was determined by visually inspecting the variances and testing correlations between ROM_max_ time points using descriptive statistics and Pearson correlation tests (*r*), respectively. These analyses determined that “compound symmetry” and “unstructured” types of covariance structures should be used in the models. In all models, “time” (pre-rehabilitation, mid-rehabilitation, and post-rehabilitation) was used as a fixed effect and “participants” was used as a random effect, while ROM_max_ was used as a dependent variable. Wilcoxon Signed-Rank tests were used to compare rectus femoris and shoulder deltoid EMG (RMS) values during the application of active *a*tDCS compared to without the application of *a*tDCS for the tDCS group, and with the application of sham *a*tDCS compared to without the application of *a*tDCS for the *Sham* group. Additional Wilcoxon Signed-Rank tests were used to compare EEG-derived BSI and clinical/functional outcomes from pre- to post-rehabilitation intervention for each group. Cohen's *d* effect sizes (*d*) were calculated for each statistical comparison. Significance was set at *p*  < 0.05. All analyses were performed with SPSS 21.0 (Statistical Package for Social Sciences, Armonk, NY, USA).

## 3. Results

### 3.1. EMG Activity

**Figure 3:**
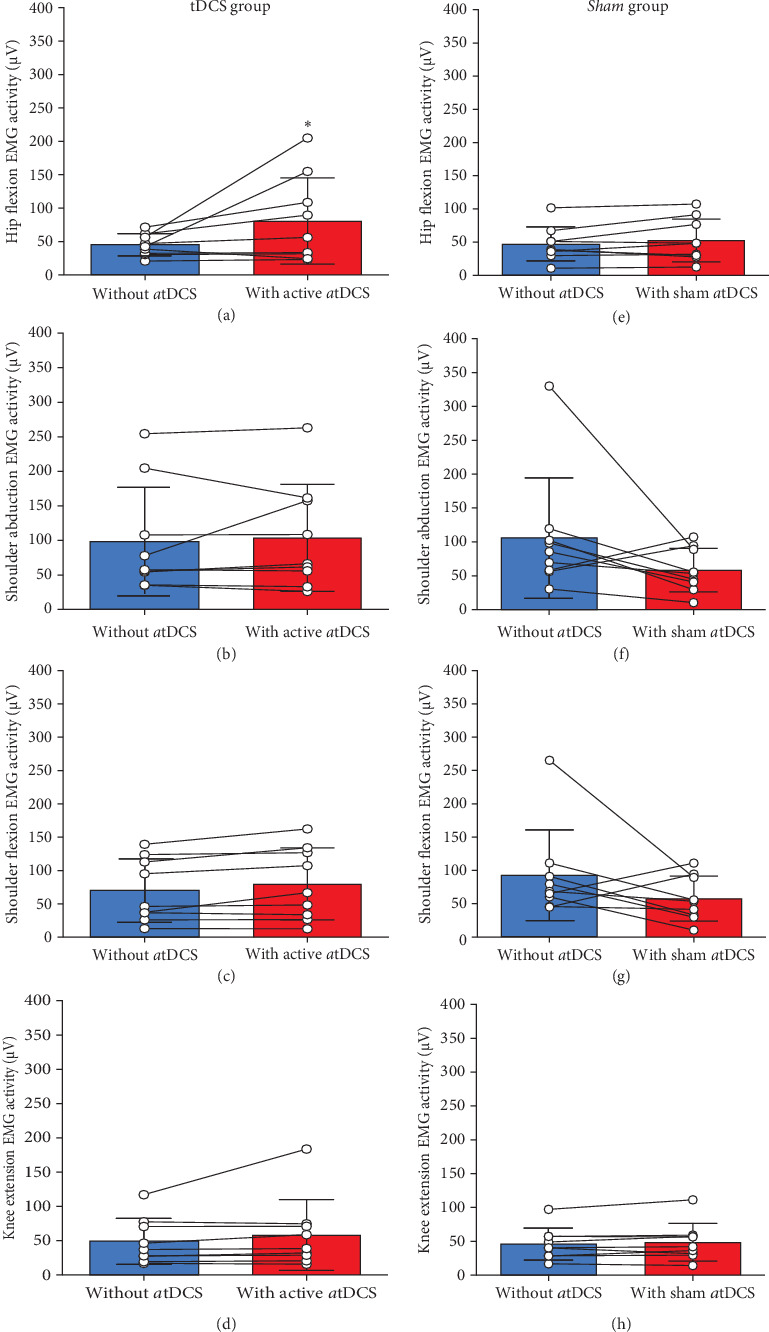
Changes in the paretic rectus femoris electromyographic (EMG) activity (root mean square calculation) during hip flexion (a and e) and knee extension (d and h), and paretic deltoid EMG activity during shoulder abduction (b and f) and shoulder flexion (c and g) maximal voluntary contractions without the application of *a*tDCS followed by with the application of active *a*tDCS on the M1 for the tDCS group, and without the application of *a*tDCS followed by with the application of sham atDCS on the M1 for the *Sham* group. Bars represent means ± SD, and white circles represent individual participant values. *⁣*^*∗*^Indicates significant difference between conditions (*p*  < 0.05).

Significant differences were evident for EMG (RMS) activity during hip flexion MVIC when participants from the tDCS group received the application of active *a*tDCS compared to without receiving *a*tDCS on the M1 in the baseline session (*Z* = −2.07, *d* = 0.77, *p*=0.04). EMG during hip flexion MVIC was 31.4% ± 26.4% (range: 3.6–72.6) greater with the application of active *a*tDCS on the M1 than without the application of *a*tDCS (Figure 3a). However, no significant differences were observed for EMG (RMS) activity during shoulder abduction (*Z* = −0.41, *d* = 0.07, *p*=0.68) (Figure 3b), shoulder flexion (*Z* = −1.95, *d* = 0.19, *p*=0.051) (Figure 3c), and knee extension (*Z* = −1.59, *d* = 0.21, *p*=0.11) (Figure 3d) MVICs when participants received active *a*tDCS compared to without receiving *a*tDCS.

**Figure 4:**
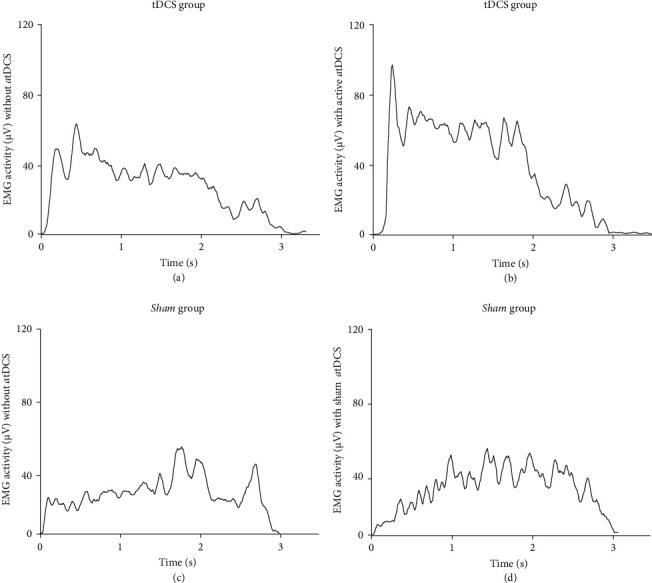
Raw traces of the paretic rectus femoris electromyographic (EMG) activity (root mean square calculation) during hip flexion maximal voluntary isometric contractions without the application of *a*tDCS (a) followed by with the application of active *a*tDCS on the M1 (b) for a single participant of the tDCS group, and without the application of *a*tDCS (c) followed by with the application of sham *a*tDCS on the M1 (d) for a single participant of the *Sham* group.

In addition, no significant differences were observed for EMG (RMS) activity during hip flexion (*Z* = −1.01, *d* = 0.19, *p*=0.31) (Figure 3e), shoulder abduction (*Z* = −1.71, *d* = 0.71, *p*=0.86) (Figure 3f), shoulder flexion (*Z* = −1.84, *d* = 0.65, *p*=0.66) (Figure 3g), and knee extension (*Z* = −1.01, *d* = 0.10, *p*=0.31) (Figure 3h) when participants from the *Sham* group received sham *a*tDCS than without receiving *a*tDCS.  Figure 4 shows hip flexion EMG (RMS) raw traces of two participants who represented the group average values of the tDCS and *Sham* groups, respectively.

### 3.2. ROM_max_

**Figure 5:**
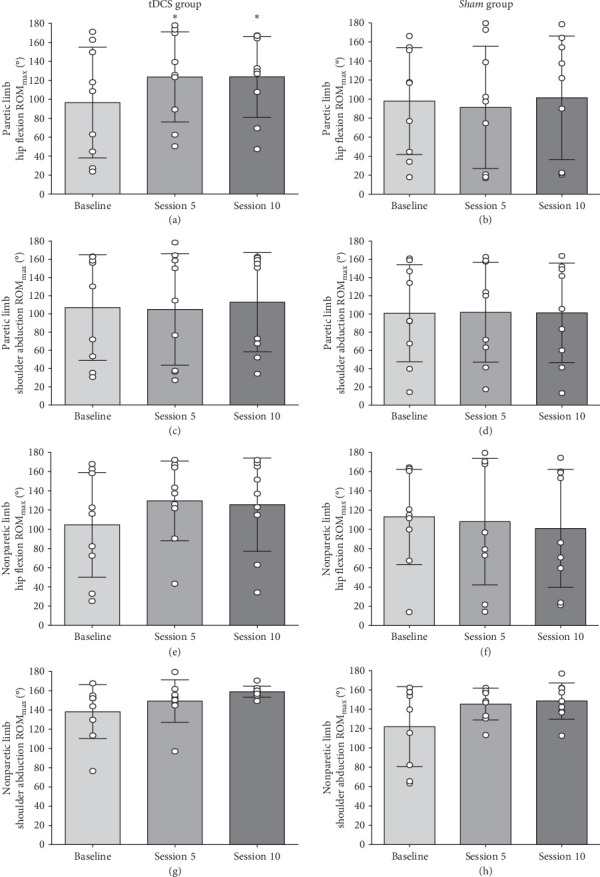
Changes in hip flexion and shoulder abduction active maximum joint range of motion (ROM_max_) from baseline (session 1) to sessions 5 (mid-rehabilitation) and 10 (post-rehabilitation) for the tDCS and *Sham* groups. Panels (a–d) refer to the paretic limb, and panels (e–h) refer to the nonparetic limb of stroke survivors. Bars represent means ± SD, and white circles represent individual participant values. *⁣*^*∗*^Indicates significant difference from baseline (*p* < 0.05).

Linear mixed models revealed significant time effects for changes in the paretic hip flexion ROM_max_ (*F*_2,16_ = 6.66, *p*=0.008) from session one (baseline) compared to session five (mid-rehabilitation) and session 10 (post-rehabilitation) for the tDCS group. Hip flexion ROM_max_ of the paretic limb at baseline was 96.6° ± 58.3° (range: 27.4° to 173.1°), and increased by 27.7% ± 24.3% from baseline to session five, and by 25.5% ± 29.6% from baseline to session 10 (*d* = 0.51–0.53, *p*  < 0.01, Figure 5a). However, no significant time effects were observed for shoulder abduction ROM_max_ (F_2,16_ = 0.60, *d* = 0.03–0.11, *p*=0.56) of the paretic limb (Figure 5c), and hip flexion (F_2,16_ = 2.73, *d* = 0.41–0.52, *p*=0.09) and shoulder abduction ROM_max_ (*F*_2,9_ = 3.06, *d* = 0.44–1.03, *p*=0.10) of the nonparetic limb (Figure 5e,g).

In addition, no significant time effects were observed for hip flexion (*F*_2,16_ = 0.45, *d* = 0.06–0.11, *p*=0.64) and shoulder abduction ROM_max_ (*F*_2,16_ = 0.02, *d* = 0.01–0.02, *p*=0.98) of the paretic limb (Figure 5b,d), and hip flexion (*F*_2,16_ = 0.58, *d* = 0.09–0.22, *p*=0.57) and shoulder abduction ROM_max_ (*F*_2,8_ = 4.54, *d* = 0.74–0.82, *p*=0.053) of the nonparetic limb for the *Sham* group (Figure 5f, h).

### 3.3. Functional Tests

Functional test results are shown in Table 2. Significant time effects were found for changes in TUG (*Z* = −2.20, *d* = 0.21, *p*=0.028) from pre- to post-intervention for the tDCS group. The time to complete the TUG test at baseline was 34.2 ± 18.2 s (range 15–70 s) and decreased by 17.4% ± 18.1% from baseline to session 10. In contrast, no significant time effects were observed for TUG for the *Sham* group (*Z* = −1.95, *d* = 0.33, *p*=0.051).

Furthermore, no significant time effects were observed for the 10-m walk test for the tDCS (*Z* = −1.63, *d* = 0.24, *p*=0.10) or *Sham* groups (*Z* = −0.89, *d* = 0.11, *p*=0.37).

### 3.4. Clinical Tests

Clinical test results are shown in Table 2. Significant time effects, indicating improvements from pre- to post-rehabilitation, were found for changes in Fugl-Meyer scale total score (tDCS group: *Z* = −2.81, *d* = 0.21, *p*=0.005; *Sham* group: *Z* = −2.21, *d* = 0.25, *p*=0.03), and Fugl-Meyer upper-extremity (tDCS group: *Z* = −2.53, *d* = 0.18, *p*=0.01; *Sham* group: *Z* = −2.01, *d* = 0.18, *p*=0.04), and lower-extremity (tDCS group: *Z* = −2.38, *d* = 0.30, *p*=0.02; *Sham* group: *Z* = −2.04, *d* = 0.33, *p*=0.03) motor subscale scores for both groups.

Similarly, significant time effects, indicating improvements from pre- to post-rehabilitation, were found for changes in the Berg Balance Scale score (tDCS group: *Z* = −2.68, *d* = 0.47, *p*=0.007; *Sham* group: *Z* = −2.37, *d* = 0.21, *p*=0.02) for both groups.

### 3.5. EEG-Derived BSI

**Figure 6:**
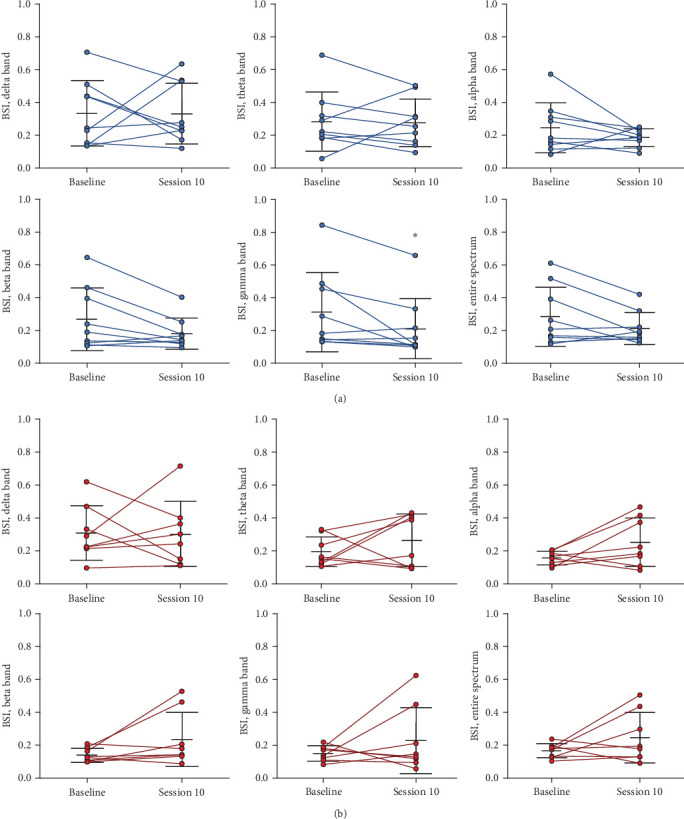
Changes in the electroencephalography (EEG)-brain symmetry index (BSI) in the delta, theta, alpha, beta and gamma bands, and across the entire spectrum, from baseline (session 1) to session 10 (post-rehabilitation) for the tDCS (a) and *Sham* (b) groups. Vertical lines represent means ± SD. Blue circles represent individual participant values of the tDCS group, and red circles represent individual participant values of the *Sham* group. *⁣*^*∗*^Indicates significant difference from baseline (*p* < 0.05).

EEG-derived BSI results are shown in Figure 6. Significant time effects were found for changes in BSI in the gamma band (*Z* = −2.07, *d* = 0.47, *p*=0.04) from pre- to post-intervention for the tDCS group. BSI in the gamma band at baseline was 0.31 ± 0.24 (range: 0.13–0.84) and decreased to 0.21 ± 0.18 (range: 0.10–0.66) in session 10, indicating increased brain symmetry. However, although a trend for decreases in BSI were observed in the alpha and higher frequency bands, no significant time effects occurred for BSI in the delta (*Z* = −0.18, *d* = 0.01, *p*=0.86), theta (*Z* = −0.53, *d* = 0.05, *p*=0.59), alpha (*Z* = −1.24, *d* = 0.52, *p*=0.21) and beta (*Z* = −1.84, *d* = 0.58, *p*=0.07) bands, nor across the entire spectrum (*Z* = −1.36, *d* = 0.50, *p*=0.17).

Furthermore, no significant time effects occurred for BSI in the delta (*Z* = 0.001, *d* = 0.04, *p*=1.00), theta (*Z* = −1.26, *d* = 0.53, *p*=0.21), alpha (*Z* = −1.26, *d* = 0.90, *p*=0.21), beta (*Z* = −1.54, *d* = 0.79, *p*=0.12), and gamma (*Z* = −0.84, *d* = 0.53, *p*=0.40) bands nor across the entire spectrum (*Z* = −1.12, *d* = 1.09, *p*=0.26) for the *Sham* group.

## 4. Discussion

In the present study, we explored the specific effects of *a*tDCS applied during XR rehabilitation exercises on cortical, neuromuscular, clinical, and functional performance outcomes of stroke survivors. We hypothesized that *a*tDCS would potentiate muscle activation, and that the 2-week rehabilitation exercise program including XR paired with *a*tDCS would lead to greater interhemispheric brain symmetry, active joint ROM and clinical/functional performance outcomes in this population. As expected, surface EMG activity was greater during hip flexion when participants from the tDCS group received the application of active *a*tDCS than without receiving *a*tDCS on the M1 (tested in the baseline session), although the same effect did not occur for the other muscle/body movements (Figures 3 and 4). Additionally, hip flexion ROM_max_ of the paretic limb increased in sessions 5 and 10 compared to baseline for the tDCS group but not for the *Sham* group (Figure 5). Participants from the tDCS group also showed a decrease in EEG-derived BSI (gamma band), indicating that greater interhemispheric brain symmetry occurred (Figure 6), and had faster results in the TUG functional test (Table 2) from before to after the rehabilitation program. However, in contrast to our hypothesis, no differences resulting from the active *a*tDCS intervention were found for upper limb active ROM_max_, and similar effects (i.e., no differences or similar improvements) were found for the other functional/clinical performance outcomes between the two groups. Thus, it seems that the application of active *a*tDCS targeted the ipsilesional M1 representation of the quadriceps, which potentiated muscle activation in the paretic rectus femoris muscle and resulted in a greater motor recovery in hip flexion movements (evidenced by ROM_max_ and TUG results) for the tDCS than for the *Sham* group. In addition, the increased brain symmetry from before to after rehabilitation for the tDCS group suggests that *a*tDCS applied during the rehabilitation program is effective in reorganizing the ipsilesional hemisphere activity after stroke.

Stroke survivors often exhibit abnormal levels of EMG activity in their paretic muscles, which may result from abnormal MU behavior, including altered recruitment patterns and compressed recruitment threshold ranges, reductions and failure to increase discharge rates during voluntary muscle contractions, and/or impaired modulation of firing rates [12, 13, 51–53]. EMG activity has also been found to be abnormally low in the paretic muscle tibialis anterior and high in the nonparetic homologous muscle during stationary locomotion activities [53]. In the present study, rectus femoris muscle activation (measured by surface EMG activity) during hip flexion MVIC of the paretic limb was on average 31% greater with active *a*tDCS than without *a*tDCS applied on the M1 for the tDCS group. In contrast, no differences were found for EMG activity when participants from the *Sham* group received sham *a*tDCS compared to not receiving *a*tDCS on the M1 (Figures 3 and 4). We are not aware of any other study that examined the effects of *a*tDCS on muscle activation of stroke survivors. However, Nistche and Paulus [54, 55], in two studies with healthy adults, reported that corticospinal excitability in the right abductor digiti minimi muscle, measured by transcranial magnetic stimulation (TMS)-induced motor evoked potential (MEP) amplitudes, increased rapidly up to 40% after short-duration *a*tDCS (5-min stimulation), and elevated to 150% above baseline values for up to 90 min after longer-duration *a*tDCS (13-min stimulation). Although it is difficult to make proper comparisons between studies because of disparate methodological approaches, our results seem to be aligned to these findings as *a*tDCS may have induced greater corticospinal excitatory responses in the ipsilesional M1 representation of the quadriceps, which increased voluntary drive during paretic hip flexion MVIC as a result of improved MU recruitment and/or discharge rates. Since the rectus femoris is the primary muscle from the quadriceps responsible for hip flexion while also assisting with synergistic knee extension actions [56], it seems that *a*tDCS was effective in particularly increasing EMG activity of this muscle in the paretic limb of stroke survivors.

Reductions in ROM_max_ have been considered as a common complication that occur in patients with chronic neurological diseases [11, 57]. In stroke patients, ROM_max_ loss may be related to inflammations to the joint capsule [11] and/or muscle spasticity of the paretic limbs [58, 59]. Previous studies have used stretching exercises for improving passive ROM_max_ (measured with handheld goniometers) of stroke patients [58, 60], but the effects of rehabilitation exercises on active ROM_max_ of the paretic limbs (measured with motion analysis systems) are poorly understood. Waldman et al. [59] found that stroke survivors who performed 18 sessions (3 times a week for 6 weeks) of controlled passive stretching and active movement exercises using a portable rehabilitation robot device increased dorsiflexion passive ROM_max_ of the paretic limb from before to after the rehabilitation program. However, active dorsiflexion ROM_max_ (measured by the same rehabilitation robotic device) was similarly improved compared to a control group who performed ankle mobility and strength instructed exercises at home for the same period of time. In contrast, in the present study, XR combined with active *a*tDCS (i.e., tDCS group) was effective in increasing hip flexion active ROM_max_ of the paretic limb by ~ 26% after only 5 daily sessions, which remained increased until the end of the 10-session rehabilitation program (Figure 5). Since active ROM_max_ changes were not seen after rehabilitation for the *Sham* group and no ROM_max_ increases were found in the shoulder abductors for any group, it seems that *a*tDCS was in fact effective in increasing neural drive to the paretic muscles responsible for hip flexion (including quadriceps rectus femoris as previously described), which allowed participants to reach greater active ranges during this movement of the paretic limb.

Conflicting results have been found by previous studies regarding the effectiveness of XR combined with *a*tDCS as a rehabilitation strategy to improve clinical and functional outcomes in stroke patients. Llorens et al. [21] found that a stroke rehabilitation program including XR paired with *a*tDCS, performed for 25 1-h sessions, was effective in improving upper limb function (measured by Fugl-Meyer upper extremity subscale, Wolf Motor Function, and Nottingham Sensory Assessment) compared to a conventional physical therapy program. In contrast, Viana et al. [28] found that both XR paired with active *a*tDCS and XR paired with sham *a*tDCS interventions were similarly effective for improving motor function measures tested in stroke survivors, including Fugl-Meyer upper extremity subscale, Wolf Motor Function test, Modified Ashworth Scale (MAS) and grip strength test. As shown in Table 2, these findings are consistent with the current study as both groups (tDCS and *Sham*) improved clinical tests (Fugl-Meyer total, upper, and lower-extremity scales, and Berg Balance Scale) similarly from before to after rehabilitation. These findings suggest that the XR rehabilitation exercise program itself, which was performed equally by both groups, was effective to improving motor function and balance of individuals after stroke. However, previous studies have suggested caution in the use of clinical scales (such as Fugl-Meyer motor scales) as main outcome measures to be compared from before to after rehabilitation programs, since these tests may be limited by a ceiling effect when stroke patients reach the maximum scores or when the scores of participants plateau (i.e., specific motor improvements may occur which are not subjectively identified by the scale used) [28, 61].

In the present study, only participants from the tDCS group significantly improved the TUG functional test (achieving on average ~ 11% faster times) from before to after the rehabilitation program, while the 10-m walk test was unchanged for both groups (Table 2). Since TUG consists of rising from a chair, walking, and returning to a seated position, the greater active hip flexion ROM_max_ of the paretic limb seen in the tDCS group during and after the rehabilitation period (Figure 5) might have improved the between-limb hip flexion movements necessary for this task. However, the same movement-specific transfer did not occur in the 10-m walk test. Although this is intriguing, the lack of improvements in this test might be explained as dynamic walking involves more complex and rapid coordinated movements of arms and legs and may lead to a greater fear of falling in stroke patients, as this test is performed for a longer distance and without having a chair to start and finish the task compared to TUG.

A disruption between the two cortical hemispheres is likely to occur after stroke due to impairment of transcallosal interhemispheric connections, which leads to hyperexcitability or disinhibition of the unaffected (nonlesioned) motor cortex [27, 62, 63]. Synder et al. [14] reported that the cortical activity network in higher frequency ranges (15–50 Hz, measured by EEG in resting state) was more asymmetric with less activity occurring in the affected hemisphere in stroke survivors compared to age-matched neurological intact adults. In addition, they found that stroke survivors' network connectivity was reduced in the alpha and beta bands (10–20 Hz) but increased in the gamma band (35–40 Hz), which suggested that disruption and alteration of cortical networks to more asymmetric, local networks, occurred after stroke. Sebastián-Romagosa et al. [19] also demonstrated that stroke survivors had significantly greater EEG-derived BSI (frequency bands ranging from 8 to 30 Hz), which indicated greater asymmetry compared to healthy adults, and that patients with the lowest BSI (greater symmetry) had better motor function in the upper extremities (measured by Fugl-Meyer upper extremity subscale). Consistent with these findings, the current study found that BSI was higher on average at baseline but decreased significantly by 72% in the gamma band after the rehabilitation program for the tDCS group. In fact, the gamma band (30–50 Hz) has been previously found as the most accurate across the entire spectrum of EEG frequency bands (1–50 Hz) to identify brain activity changes during a virtual reality hand motor task combined with the application of active *a*tDCS on the motor cortex of stroke survivors [64]. Our findings were also followed by a trend of nonsignificant decreases in BSI in other frequency bands. In contrast, the same effect did not occur for the *Sham* group (Figure 6). Previous pharmacological studies have shown that *a*tDCS applied on the M1 may modulate the conductivity of sodium and calcium channels, and lead to alterations on the N-methyl-D-aspartate (NMDA)-receptor activation, which causes neuronal membrane depolarization and induces short- and long-lasting changes in neuroplasticity [65, 66]. Thus, the significant decrease in EEG-derived BSI in the gamma frequency band seen in the tDCS group suggests that active *a*tDCS applied on the ipsilesional motor cortex of stroke survivors may be responsible for balancing excitatory and inhibitory processes between hemispheres, which in turn restores interhemispheric symmetry and leads to plastic structural changes within the affected side of the brain.

We acknowledge that several factors should be considered when interpreting the findings of the present study. First, not all participants that were recruited suffered stroke in the same cortical region, which may have resulted in different levels of hemiparesis and consequently influenced the large inter-individual variability of the data. Since most of the data presented high inter-individual variability, which likely contributed to the deviations from normality, we utilized alternative statistical analyses such as non-parametric tests and linear mixed models to compare changes in the dependent variables from pre- to post-rehabilitation. In particular, linear mixed models with a repeated measures design were applied when the analyses involved comparing data in more than two time points, as unlike repeated measures ANOVA these models do not depend on limited assumptions about the variance-covariance matrix and are less dependent on the homogeneity of variances [49]. However, these statistical tests did not allow the assessment of additional interactions between factors or direct post-hoc statistical analyses. In addition, due to the increased data variability and limited sample size of the study, we did not perform further adjustments for multiple comparisons, although the effect sizes of each statistical comparison were reported. Thus, more definitive conclusions about the role of *a*tDCS applied during XR rehabilitation may not be possible at this stage. Second, pre-, mid-, and post-rehabilitation measurements were included before or after the first, fifth and tenth sessions (and not on separate days) from the rehabilitation program, since the inclusion of additional days could compromise the compliance of stroke survivor participants to the rehabilitation program and increase the risk of dropouts in the study. Thus, resting measurements (EEG and ROM) were always performed prior to the start of these training sessions. In addition, clinical/functional measurements were included before the first session and at the end of the tenth session for an overall understanding of the changes in motor function after the rehabilitation program, which may have increased the risk of neuromuscular fatigue affecting the results of these variables. However, to minimize this risk, enough rest time (25–30 min) was given between the end of the tenth rehabilitation session and the post-rehabilitation clinical/functional measurements. Third, although EMG results indicate that the M1 representation of the rectus femoris muscle was more activated with active *a*tDCS, it was not possible to determine the precise cortical location in which the stimulation would be delivered to target a specific muscle. The inclusion of TMS measures may further explore the exact hotspot in which *a*tDCS should be positioned to elicit consistent corticospinal responses for each specific muscle and should be considered in future studies. Finally, in the present study, EMG (RMS) values were not further normalized by MVIC or peak EMG values since methodological limitations exist when normalizing muscle activation levels of stroke survivors, as they commonly present increased muscle weakness or muscle spasticity [41, 42]. An alternative method for normalizing rectus femoris EMG could be using electrical nerve stimulation to induce maximal compound muscle action potentials (M_MAX_) in the muscle. However, previous studies have also reported limitations with this normalization method, as stroke survivors may have poor EMG relative to *M*_MAX_ values in the paretic muscles as a result of their increased levels of atrophy and denervation of muscle fibers [67], and that this method does not provide any significant advantage compared to not normalizing EMG activity in this population [68].

In conclusion, the findings of the present study showed that a rehabilitation program that included active *a*tDCS applied during XR exercises was effective for improving EEG-derived BSI, hip flexion active ROM_max_, and TUG functional performance of stroke survivors. Furthermore, *a*tDCS applied on the M1 boosted muscle activation of the paretic rectus femoris measured by EMG. Therefore, it seems that *a*tDCS targeted the ipsilesional M1 representation of the quadriceps, which potentiated muscle activation in the paretic rectus femoris muscle and resulted in greater motor recovery in hip flexion movements for the tDCS group. The decreased BSI observed in the tDCS group also suggests that *a*tDCS applied during rehabilitation is effective in reorganizing the ipsilesional hemisphere activity after stroke.

## Figures and Tables

**Table 1:** Mean ± SD of age, months post-stroke, modified rankin scale, and extremities affected by hemiparesis for individuals in the tDCS and *Sham* groups.

		tDCS group (*n* = 10)	*Sham* group (*n* = 9)	*p*
Age (years)	Mean (SD)	62.4 ± 10.8	57.5 ± 15.5	0.70
Months post stroke (months)	Mean (SD)	33.9 ± 22.7	21.9 ± 20.9	0.25
Modified rankin scale	Mean (SD)	2.2 ± 0.8	2.1 ± 1.1	0.73
Hemiparesis	—	*n* = 3 right in the right extremities *n* = 7 in the left extremities	*n* = 3 right in the right extremities *n* = 6 in the left extremities	—

*Note:* No significant effects of group were found for any variable (*p*  > 0.05).

**Table 2:** Changes (mean ± standard deviation) in the time up and go (TUG), 10-m walk, Fugl-Meyer upper and lower extremity subscale scores, Fugl-Meyer total score, and Berg Balance Scale from baseline (pre-rehabilitation) to session 10 (post-rehabilitation) for the tDCS and *Sham* groups.

		tDCS group		*Sham* group	
		Baseline	Session 10	Baseline	Session 10
TUG (s)	*Mean ± SD*	34.2 ± 18.2	30.6 ± 16.3*⁣*^*∗*^	28.4 ± 16.9	23.3 ± 13.8
	*P*	0.03		>0.05	
10-m walk	*Mean ± SD*	58.5 ± 35.5	50.9 ± 26.9	23.6 ± 21.7	21.7 ± 9.2
(s)	*P*	0.10		0.37	
Fugl-Meyer	*Mean ± SD*	37.5 ± 21.9	41.3 ± 20.5*⁣*^*∗*^	28.0 ± 16.8	31.1 ± 17.5*⁣*^*∗*^
(upper extremity score)	*P*	0.01		0.04	
Fugl-Meyer	*Mean ± SD*	24.7 ± 7.0	26.8 ± 6.9*⁣*^*∗*^	21.3 ± 8.1	24.0 ± 8.3*⁣*^*∗*^
(lower extremity score)	*P*	0.02		0.03	
Fugl-Meyer	*Mean ± SD*	62.2 ± 28.4	68.1 ± 26.8*⁣*^*∗*^	49.3 ± 22.8	55.1 ± 23.9*⁣*^*∗*^
(Total score)	*P*	<0.01		0.03	
Berg Balance Scale	*Mean ± SD*	43.9 ± 8.6	47.7 ± 7.6*⁣*^*∗*^	39.8 ± 17.1	43.4 ± 17.6*⁣*^*∗*^
(Score)	*P*	<0.01		0.02	

*⁣* ^*∗*^Indicates significant difference compared with the pre-rehabilitation (baseline) values (*p*  < 0.05).

## Data Availability

The data that supports the findings of this study are available from the corresponding author upon reasonable request.
